# Coronavirus conspiracy suspicions, general vaccine attitudes, trust and coronavirus information source as predictors of vaccine hesitancy among UK residents during the COVID-19 pandemic

**DOI:** 10.1017/S0033291721001434

**Published:** 2021-04-12

**Authors:** Daniel Allington, Siobhan McAndrew, Vivienne Moxham-Hall, Bobby Duffy

**Affiliations:** 1 Digital Humanities, King's College London, London WC2R 2LS, UK; 2 School of Sociology, Politics, and International Studies, University of Bristol, Bristol BS8 1TU; 3 Policy Institute, King's College London, London WC2R 2LS

**Keywords:** Coronavirus, COVID-19, Vaccine hesitancy, Conspiracy beliefs, Conspiracy theories

## Abstract

**Background:**

Vaccine hesitancy presents an obstacle to the campaign to control COVID-19. It has previously been found to be associated with youth, female gender, low income, low education, low medical trust, minority ethnic group membership, low perceived risk from COVID-19, use of certain social media platforms and conspiracy beliefs. However, it is unclear which of these predictors might explain variance associated with others.

**Methods:**

An online survey was conducted with a representative sample of 4343 UK residents, aged 18–75, between 21 November and 21 December 2020. Predictors of vaccine hesitancy were assessed using linear rank-order models.

**Results:**

Coronavirus vaccine hesitancy is associated with youth, female gender, low income, low education, high informational reliance on social media, low informational reliance on print and broadcast media, membership of other than white ethnic groups, low perceived risk from COVID-19 and low trust in scientists and medics, as well as (to a much lesser extent) low trust in government. Coronavirus conspiracy suspicions and general vaccine attitudes appear uniquely predictive, jointly explaining 35% of variance. Following controls for these variables, effects associated with trust, ethnicity and social media reliance largely or completely disappear, whereas the effect associated with education is reversed.

**Conclusions:**

Strengthening positive attitudes to vaccination and reducing conspiracy suspicions with regards to the coronavirus may have a positive effect on vaccine uptake, especially among ethnic groups with heightened vaccine hesitancy. However, vaccine hesitancy associated with age and gender does not appear to be explained by other predictor variables tested here.

## Introduction

Vaccine hesitancy, or ‘delay in acceptance or refusal of vaccination despite availability of vaccination services’ (MacDonald & Sage Working Group on Vaccine Hesitancy, 2015, p. 4163), has been identified as one of the greatest threats to public health at a global level (WHO, 2019). A refusal rate of more than 10% is estimated to be sufficient to undermine the population benefits of vaccination against SARS-CoV-2, the novel coronavirus which causes COVID-19 (DeRoo, Pudalov, & Fu, 2020).

Vaccine hesitancy appears to be a particular problem for the developed world. For example, negative views of vaccines are far higher in both Western and Eastern Europe than they are in South Asia and Eastern Africa (Wellcome, 2019, pp. 110–111). Some argue that vaccine hesitancy in the developed world is currently driven by the circulation of misinformation on social media (Rochel de Camargo, 2020, p. 3; see Suarez-Lledo & Alvarez-Galvez, 2021 for a systematic review of studies of medical misinformation on social media), which could explain why under-vaccination and delayed vaccination have been rising for some time in the USA, and reached a level that has been described as a ‘cultural epidemic’ in Europe (McIntosh, Janda, Ehrich, Pettoello-Mantovani, & Somekh, 2016; Salmon, Dudley, Glanz, & Omer, 2015). Many studies have found a link between vaccine hesitancy and exposure to online anti-vaccination materials, as well as to the conspiracy theories that they so often promote (Ahmed, Quinn, Hancock, Freimuth, & Jamison, 2018; Dunn, Leask, Zhou, Mandl, & Coiera, 2015; 2017; Lyons, Merola, & Reifler, 2019; Wilson & Wiysonge, 2021), and associations between coronavirus vaccine hesitancy, coronavirus conspiracy beliefs and/or use of social media or non-mainstream media as an information source have been reported (Allington, Duffy, Wessely, Dhavan, & Rubin, 2020; 2021; Bertin, Nera, & Delouvée, 2020; Freeman et al., 2020; Jennings et al., 2021; McAndrew & Allington, 2020; Murphy, Vallières, Bentall, Shevlin, & McBride, 2020; Romer & Jamieson, 2020), with one recent study finding among people whose news diet is dominated by social media an association between conspiracy mentality and the intention to discourage coronavirus vaccination (Chadwick et al., 2021). There is also experimental support for a causal relationship between exposure to anti-vaccination materials (including conspiracy theories) and reduced intention to vaccinate (Betsch, Renkewitz, Betsch, & Ulshöfer, 2010; Chen, Zhang, Young, Wu, & Zhu, 2020; Jolley & Douglas, 2014). The largest experimental study found exposure to coronavirus- and vaccine-related misinformation to reduce the proportion of participants who say that they would ‘definitely’ be vaccinated against the coronavirus by 6.2 percentage points in the UK and 6.4 percentage points in the USA (Loomba, de Figueiredo, Piatek, de Graaf, & Larson, 2021).

However, there are many other variables that might explain vaccine hesitancy. General vaccine attitudes have been found to predict coronavirus vaccine refusal in the USA, especially among women and African Americans (Callaghan et al., 2021), and many studies have found a relationship between trust and vaccine hesitancy (Dubé, Gagnon, Nickels, Jeram, & Schuster, 2014; Hornsey, Lobera, & Díaz-Catalan, 2020; Mills, Jadad, Ross, & Wilson, 2005; Quinn, Jamison, An, Hancock, & Freimuth, 2019; Wilder-Smith & Qureshi, 2020). It is also plausible that scientific, medical and political mistrust might drive both vaccine hesitancy and conspiracy beliefs. For example, both the heightened rates of HIV conspiracy belief and the lower rates of HIV-protective behaviours within the African American community have been attributed to mistrust triggered by the experience of racism (Ball, Lawson, & Alim, 2013, p. 4). Some anti-vaccine campaigners have adopted a strategy of ‘framing COVID-19 vaccination in terms of past medical abuses against minority groups’ (Callaghan et al., 2021, p. 2), and there is qualitative evidence to suggest that coronavirus vaccine hesitancy among African Americans may be driven by mistrust in the medical establishment and by perceptions of racism in the political system (Momplaisir et al., 2021). Similarly, some experts have attributed heightened levels of coronavirus misinformation acceptance and coronavirus vaccine hesitancy within some minority ethnic communities in the UK to mistrust driven by experiences of racism, discrimination and exclusion (Razai, Osama, McKechnie, & Majeed, 2021, p. 1).

The current study accordingly tests for the possible impact on coronavirus vaccine hesitancy in the UK of conspiracy beliefs with regards to the coronavirus, general vaccine attitudes, risk perceptions, informational reliance on social media and trust in scientists, healthcare professionals and the government. Other factors which recent British studies have found to be associated with coronavirus vaccine hesitancy include youth, female gender, minority ethnic group membership, low education and low income, with the perception of the coronavirus as a personal threat being associated with lower levels of hesitancy (Allington, McAndrew, Moxham-Hall, & Duffy, 2021; Freeman et al., 2020; Jennings et al., 2021); all of these are therefore also tested for. By using a large representative sample and constructing a variety of multivariate models, this study contributes to knowledge at once by attempting to replicate the findings of previous studies and by attempting to explain and establish the relative importance of the predictors through controls.

## Hypotheses

The study was designed to test the hypothesis that coronavirus vaccine hesitancy will have:

(1) A negative relationship with age
(2) A positive relationship with female gender
(3) A positive relationship with membership of other than white ethnic groups
(4) A negative relationship with level of education
(5) A negative relationship with household income
(6) A negative relationship with reliance on legacy media as a source of information about coronavirus
(7) A positive relationship with reliance on social media as a source of information about coronavirus
(8) A negative relationship with perceived risk from the coronavirus, with regards to:
  (a) The self
  (b) People in the UK
  (c) People elsewhere in the world
(9) A negative relationship with trust in:
  (a) The national government
  (b) Scientists working in the academic sector
  (c) Scientists working in the private sector
  (d) Medical professionals
(10) A negative relationship with general attitudes to vaccination
(11) A positive relationship with conspiracy suspicions with regards to the coronavirus

Hypotheses 6–11 were tested both before and after controlling for the demographic variables mentioned in hypotheses 1–5. For analytic purposes, all ethnic groups were aggregated into two categories: white and other than white. Education was operationalised as highest completed level of education, which was measured on an ordinal scale ranging from primary school to postgraduate qualifications or their equivalent. In view of Quinn *et al*.'s (2019) argument that it is important to distinguish general attitudes from those relating to a specific vaccine, it is emphasised that hypothesis 10 relates to attitudes to vaccination in general (and not to coronavirus vaccination in particular), and hypothesis 11 relates to conspiracy suspicions with regards to the coronavirus itself (rather than coronavirus vaccination), while the dependent variable in all cases is hesitancy with regards to coronavirus vaccination.

## Methodology

### Questionnaire

Key questionnaire items are presented in Table 1. Each respondent received one of two versions of the vaccine hesitancy question, at random. The first version asked ‘If a vaccine for coronavirus becomes available, how likely or unlikely would you personally be to get the vaccine?’ The second asked ‘If a vaccine for coronavirus becomes available, how likely or unlikely would you personally be to get the vaccine if you were offered it?’ This was done because of concerns raised that the question might be conflating likelihood of accepting the vaccine with likelihood of being offered it (see Full Fact, 2021).

**Table 1.** Key questionnaire items

Variable	Text	Answers
Hesitancy	If a vaccine for coronavirus becomes available, how likely or unlikely would you personally be to get the vaccine/if you were offered it?	Certain, Very likely, Fairly likely, Not very likely, Not at all likely, Definitely not, Don't know
Legacy med.	Please tell us how much of what you know about coronavirus, if anything, comes from…: TV and radio broadcasters (including through their websites and online)	A great deal, A fair amount, Not very much, Nothing at all, Don't know
Legacy med.	Please tell us how much of what you know about coronavirus, if anything, comes from…: Newspapers and magazines (including through their websites and online)	A great deal, A fair amount, Not very much, Nothing at all, Don't know
Social med.	Please tell us how much of what you know about coronavirus, if anything, comes from…: YouTube	A great deal, A fair amount, Not very much, Nothing at all, Don't know
Social med.	Please tell us how much of what you know about coronavirus, if anything, comes from…: Facebook	A great deal, A fair amount, Not very much, Nothing at all, Don't know
Social med.	Please tell us how much of what you know about coronavirus, if anything, comes from…: WhatsApp	A great deal, A fair amount, Not very much, Nothing at all, Don't know
Social med.	Please tell us how much of what you know about coronavirus, if anything, comes from…: Twitter	A great deal, A fair amount, Not very much, Nothing at all, Don't know
Social med.	Please tell us how much of what you know about coronavirus, if anything, comes from…: Instagram	A great deal, A fair amount, Not very much, Nothing at all, Don't know
Risk: pers.	To what extent, if at all, do you think the coronavirus poses a risk to…?: Yourself personally	A very high risk, A fairly high risk, Not a very high risk, No risk at all, Don't know
Risk: UK	To what extent, if at all, do you think the coronavirus poses a risk to…?: People in the UK	A very high risk, A fairly high risk, Not a very high risk, No risk at all, Don't know
Risk: world	To what extent, if at all, do you think the coronavirus poses a risk to…?: People in other countries	A very high risk, A fairly high risk, Not a very high risk, No risk at all, Don't know
Trust: gov.	To what extent, if at all, do you trust the following?: The UK Government	A great deal, A fair amount, Not very much, Not at all, Don't know
Trust: sci. uni.	To what extent, if at all, do you trust the following?: Scientists working at universities in the UK	A great deal, A fair amount, Not very much, Not at all, Don't know
Trust: sci. com.	To what extent, if at all, do you trust the following?: Scientists working at private companies in the UK	A great deal, A fair amount, Not very much, Not at all, Don't know
Trust: med.	To what extent, if at all, do you trust the following?: Doctors and nurses in the UK	A great deal, A fair amount, Not very much, Not at all, Don't know
Vacc. att.	Thinking firstly about vaccinations in general, to what extent, if at all, do you agree or disagree with the following?: Vaccines are important for children to have	Strongly agree, Tend to agree, Neither agree nor disagree, Tend to disagree, Strongly disagree, Don't know
Vacc. att.	Thinking firstly about vaccinations in general, to what extent, if at all, do you agree or disagree with the following?: Vaccines are safe	Strongly agree, Tend to agree, Neither agree nor disagree, Tend to disagree, Strongly disagree, Don't know
Vacc. att.	Thinking firstly about vaccinations in general, to what extent, if at all, do you agree or disagree with the following?: Vaccines are effective	Strongly agree, Tend to agree, Neither agree nor disagree, Tend to disagree, Strongly disagree, Don't know
Con. susp.	To what extent, if at all, do you agree or disagree with the following statements?: The real truth about coronavirus is being kept from the public	Strongly agree, Tend to agree, Neither agree nor disagree, Tend to disagree, Strongly disagree, Don't know
Con. susp.	To what extent, if at all, do you agree or disagree with the following statements?: People need to wake up and start asking questions about coronavirus	Strongly agree, Tend to agree, Neither agree nor disagree, Tend to disagree, Strongly disagree, Don't know
Con. susp.	To what extent, if at all, do you agree or disagree with the following statements?: Legitimate questions about coronavirus are being suppressed by the government, the media, and academia	Strongly agree, Tend to agree, Neither agree nor disagree, Tend to disagree, Strongly disagree, Don't know
Con. susp.	To what extent, if at all, do you agree or disagree with the following statements?: Reporters, scientists, and government officials are involved in a conspiracy to cover up important information about coronavirus	Strongly agree, Tend to agree, Neither agree nor disagree, Tend to disagree, Strongly disagree, Don't know
Con. susp.	To what extent, if at all, do you agree or disagree with the following statements?: An impartial, independent investigation of coronavirus would show once and for all that we've been lied to on a massive scale	Strongly agree, Tend to agree, Neither agree nor disagree, Tend to disagree, Strongly disagree, Don't know

Questionnaire items used to assess vaccine attitudes were drawn directly from the Wellcome Global Monitor survey (Wellcome, 2019, Q24, Q25 and Q26), whereas items used to assess conspiracy suspicions were adapted from the Flexible Inventory of Conspiracy Suspicions (Wood, 2016). Items used to assess informational reliance on various media sources were drawn from an earlier study of conspiracy beliefs (Allington et al., 2020). Items used to assess trust were adapted from the Wellcome Global Monitor Survey (Wellcome, 2019, Q11B, Q11E, Q14A and Q15a).

### Ethical statement

Data collection followed ethical and data protection procedures at the University of Bristol, King's College London and Ipsos MORI. Prior to commencing the study, participants were informed that they were taking part in coronavirus-related research led by the University of Bristol, and were provided with the principal investigator's name and contact details.

### Data collection

Data collection was carried out online by Ipsos MORI, and ran from 21 November to 21 December 2020. The data collection formed part of a longitudinal study in which each wave involved collection from a stratified random sample of members of a recruited panel of UK adults. As the current sample included recontactees from two previous waves, and as there was additional recruitment from other panels where responses within certain strata were inadequate to produce representativeness, it is not a probability sample. The panel was stratified for representativeness of the UK population on age, gender, region, and working status. Demographic weights were calculated post-collection by Ipsos MORI on the basis of education and geographical region and of gender interlocked with age, NRS social grade and working status. The sample was designed and weighted for demographic representativeness and is treated as equivalent to a random sample below. In total, 4343 individuals gave consent to take part and completed the questionnaire.

### Descriptive statistics

For descriptive statistics, see Table 2.^†^^1^ The 12 respondents who stated that they preferred to identify their gender in another way than as male or female are included neither in the ‘female’ nor in the ‘male’ column in Table 2; for analytic purposes, they were grouped together with males in a single non-female category. The 55 respondents who did not provide information on their ethnicity are included neither in the ‘white’ nor in the ‘other’ column; for analytic purposes, they were treated as missing data for all calculations involving ethnicity.

**Table 2.** Descriptive statistics

	Age		Gender		Ethnic group		Education		Household income		Vaccination likelihood	
	*M*	s.d.	Female	Male	White	Other	Degree	Non-degree	<£25 000	£25 000+	Cert./v. likely	Other
Unweighted	46.46	15.73	50	49	91	8	45	55	39	61	55	45
Weighted	46.23	15.87	51	49	91	7	31	69	42	58	54	46

The sample appears broadly representative of the UK population on gender, age and minority ethnic group membership, with over-representation of highly-educated individuals. Just over half the respondents stated that they were ‘certain’ or ‘very likely’ to be vaccinated.

### Key measures

In total, 2174 respondents were asked the shorter version of the vaccine hesitancy question, and 2169 were asked the longer version of the question. Welch's unequal variances *t* test on ranks confirms that there was no significant difference in answers, *t*_(3962.18)_ = 0.46, *p* = 0.648, and the items were accordingly combined to create a vaccine hesitancy indicator for our analyses, with lower expressed likelihood interpreted as greater hesitancy.

Rank-order correlations and Guttman's lambda 6 were used to assess internal reliability of scales and implied scales. Answers to items used to assess conspiracy suspicions had a mean correlation of *r*_s_ = 0.67, *λ*_6_ = 0.90. Answers to questions used to assess attitudes to vaccines in general had a mean correlation of *r*_s_ = 0.69, *λ*_6_ = 0.83. Answers to questions used to assess informational reliance on social media had a mean correlation of *r*_s_ = 0.49, *λ*_6_ = 0.80. Answers to the two questions used to assess informational reliance on the legacy media were positively correlated, *r*_s_ = 0.22. Answers to the three questions used to assess risk perceptions with regards to the coronavirus had a mean correlation of *r*_s_ = 0.56, which would have equated to *λ*_6_ = 0.77. However, these were not aggregated. Answers to the four questions used to assess trust had a mean correlation of *r*_s_ = 0.38, which would have equated to *λ*_6_ = 0.69. Again, these were not aggregated.

For all analytic purposes, ‘don't know’ and ‘prefer not to answer’ responses were treated as missing data. Altogether, 378 respondents answered ‘don't know’ to the question used to assess vaccine hesitancy.

### Analytic methodology

Analysis was carried out primarily through the construction and examination of a series of linear rank-order models. Models of this type, often described as rank regression models, involve applying the rank transformation to response and predictor variables and then fitting a linear model to the ranks using the ordinary least squares method. Models of this kind were initially proposed for modelling monotonic but non-linear relationships between continuous variables (Iman & Conover, 1979), and are widely recommended for dealing with outliers (Chen, Tang, Lu, & Tu, 2014). As the nonparametric equivalent of linear modelling, they have also been argued to be appropriate for ordinal data while requiring fewer assumptions than ordered logit or probit models (Fu, Wang, & Liu, 2012). Because they can accommodate ordinal predictors, linear rank-order models enable the preservation of more information than would be the case for logit or probit models. Moreover, unlike such models, they permit the calculation of *r*^2^, which provides a meaningful and intuitively interpretable metric of comparison between competing models. Finally, they provide an intuitive measure of effect size: a coefficient of 1.0 for an independent variable corresponds to an average increase of one rank in the dependent variable for each rank by which the independent variable increases.

Some scholars have argued that linear rank-order models may produce higher type I error rates (Headrick & Rotou, 2001). For this reason, a parallel set of logit models was created using the same variables, albeit with ordinal variables dichotomised and with continuous variables standardised. Risk, trust and level of education were dichotomised by coding the top two levels (equating to ‘A fairly high risk’ and ‘A very high risk’ for risk, ‘A fair amount’ and ‘A great deal’ for trust, and undergraduate and postgraduate degrees or their equivalent for education) as 1 and the others as 0. Vaccine hesitancy was dichotomised by coding ‘Certain’ or ‘Very likely’ as 0 and anything else as 1. Household income was dichotomised by coding the median category of £25 000–£34 999 and anything above it as 1 and everything below it as 0. Demographic weights were applied in both linear rank-order models and logit models.

A matrix of unweighted rank-order correlation coefficients is additionally provided, both in the interest of transparency and as a form of exploratory analysis.

## Findings

### Correlations

**Fig. 1.**
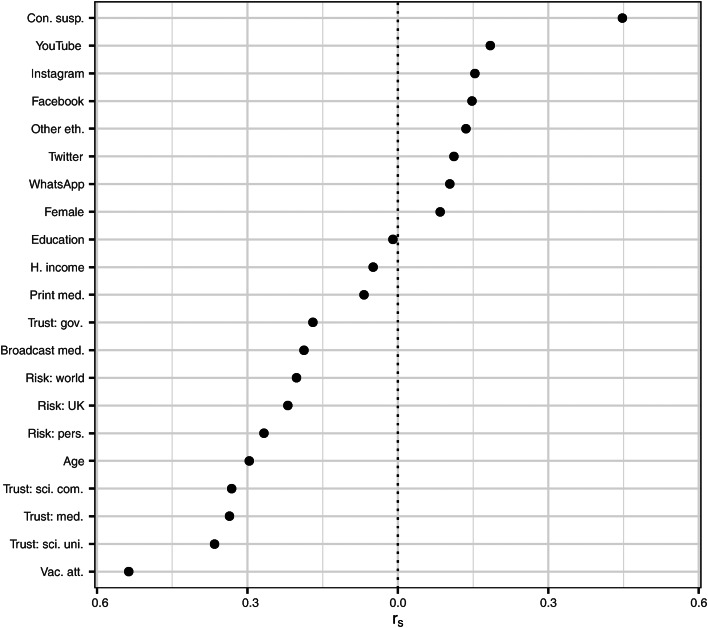
Rank-order correlations with vaccine hesitancy.

Rank-order correlations between key variables are presented in Table 3. Rank-order correlations between coronavirus vaccine hesitancy and all other variables are visualised in Fig. 1. Information source variables are unaggregated, as there is clear interest in comparing correlations both for print and broadcast media and for individual social media platforms. Informational reliance on broadcast media has a stronger negative correlation with vaccine hesitancy than does informational reliance on print media (*r*_s_ = −0.19 as opposed to *r*_s_ = −0.07), and whereas informational reliance on all social media platforms is positively correlated with vaccine hesitancy, this correlation is strongest with regards to Facebook and YouTube (*r*_s_ = 0.15 and *r*_s_ = 0.18, respectively). However, the strongest positive correlate of vaccine hesitancy is the aggregate measure of conspiracy suspicions with regards to the coronavirus (*r*_s_ = 0.45), and the strongest negative correlates are positive attitude towards vaccines in general (*r*_s_ = −0.54) and perceived risk posed by the coronavirus (especially personal risk, *r*_s_ = −0.27). After these, the strongest associations were the negative correlation with trust in scientists working in universities (*r*_s_ = −0.37) and for companies (*r*_s_ = −0.33) and with trust in medical professionals (*r*_s_ = −0.34); the negative correlation with trust in government was much weaker (*r*_s_ = −0.17).

**Table 3.** Rank-order correlations

Variable	1	2	3	4	5	6	7	8	9	10	11	12	13	14	15	16	17	18	19	20	21	22
1. Age		−0.03	−0.20	−0.23	−0.18	0.11	−0.03	−0.24	−0.30	−0.35	−0.32	−0.27	0.22	0.06	0.06	0.08	0.05	0.06	0.13	0.00	−0.09	−0.30
2. Female	−0.03		−0.01	−0.01	−0.11	−0.03	−0.01	0.06	−0.09	−0.01	−0.09	−0.02	0.00	0.11	0.08	0.02	−0.01	0.00	−0.01	−0.03	0.05	0.08
3. Other eth.	−0.20	−0.01		0.10	0.04	−0.03	0.06	0.06	0.19	0.16	0.10	0.20	0.00	0.00	0.01	0.00	−0.05	−0.05	−0.07	−0.09	0.11	0.14
4. Education	−0.23	−0.01	0.10		0.34	0.01	0.12	−0.01	0.07	0.08	0.13	0.10	−0.07	−0.06	−0.07	−0.06	0.11	0.05	−0.02	0.16	−0.15	−0.01
5. H. income	−0.18	−0.11	0.04	0.34		0.06	0.11	−0.01	0.02	0.10	0.10	0.10	−0.08	−0.07	−0.08	0.08	0.09	0.10	0.03	0.12	−0.08	−0.05
6. Broadcast med.	0.11	−0.03	−0.03	0.01	0.06		0.22	0.00	−0.03	−0.01	−0.01	0.00	0.11	0.14	0.15	0.19	0.23	0.23	0.22	0.15	−0.13	−0.19
7. Print med.	−0.03	−0.01	0.06	0.12	0.11	0.22		0.14	0.17	0.19	0.17	0.17	0.05	0.08	0.10	0.07	0.11	0.13	0.08	0.10	−0.03	−0.07
8. Facebook	−0.24	0.06	0.06	−0.01	−0.01	0.00	0.14		0.45	0.50	0.40	0.45	0.06	0.03	0.04	0.06	−0.04	−0.01	−0.06	−0.12	0.25	0.15
9. YouTube	−0.30	−0.09	0.19	0.07	0.02	−0.03	0.17	0.45		0.54	0.46	0.51	0.00	−0.03	−0.01	0.04	−0.09	−0.03	−0.13	−0.13	0.24	0.18
10. Instagram	−0.35	−0.01	0.16	0.08	0.10	−0.01	0.19	0.50	0.54		0.55	0.59	0.03	0.03	0.04	0.10	−0.05	0.02	−0.08	−0.09	0.21	0.15
11. Twitter	−0.32	−0.09	0.10	0.13	0.10	−0.01	0.17	0.40	0.46	0.55		0.45	0.01	0.02	0.02	−0.01	−0.01	0.01	−0.05	−0.01	0.11	0.11
12. WhatsApp	−0.27	−0.02	0.20	0.10	0.10	0.00	0.17	0.45	0.51	0.59	0.45		0.05	0.04	0.04	0.09	−0.03	0.03	−0.05	−0.06	0.18	0.10
13. Risk: pers.	0.22	0.00	0.00	−0.07	−0.08	0.11	0.05	0.06	0.00	0.03	0.01	0.05		0.47	0.41	0.10	0.13	0.14	0.12	0.08	−0.05	−0.27
14. Risk: UK	0.06	0.11	0.00	−0.06	−0.07	0.14	0.08	0.03	−0.03	0.03	0.02	0.04	0.47		0.80	0.08	0.20	0.18	0.22	0.13	−0.15	−0.22
15. Risk: world	0.06	0.08	0.01	−0.07	−0.08	0.15	0.10	0.04	−0.01	0.04	0.02	0.04	0.41	0.80		0.09	0.22	0.19	0.23	0.14	−0.14	−0.20
16. Trust: gov.	0.08	0.02	0.00	−0.06	0.08	0.19	0.07	0.06	0.04	0.10	−0.01	0.09	0.10	0.08	0.09		0.22	0.35	0.17	0.11	−0.19	−0.17
17. Trust: sci. uni.	0.05	−0.01	−0.05	0.11	0.09	0.23	0.11	−0.04	−0.09	−0.05	−0.01	−0.03	0.13	0.20	0.22	0.22		0.61	0.53	0.42	−0.37	−0.37
18. Trust: sci. com.	0.06	0.00	−0.05	0.05	0.10	0.23	0.13	−0.01	−0.03	0.02	0.01	0.03	0.14	0.18	0.19	0.35	0.61		0.41	0.35	−0.29	−0.33
19. Trust: med.	0.13	−0.01	−0.07	−0.02	0.03	0.22	0.08	−0.06	−0.13	−0.08	−0.05	−0.05	0.12	0.22	0.23	0.17	0.53	0.41		0.36	−0.29	−0.34
20. Vac. att.	0.00	−0.03	−0.09	0.16	0.12	0.15	0.10	−0.12	−0.13	−0.09	−0.01	−0.06	0.08	0.13	0.14	0.11	0.42	0.35	0.36		−0.46	−0.54
21. Con. susp.	−0.09	0.05	0.11	−0.15	−0.08	−0.13	−0.03	0.25	0.24	0.21	0.11	0.18	−0.05	−0.15	−0.14	−0.19	−0.37	−0.29	−0.29	−0.46		0.45
22. Hesitancy	−0.30	0.08	0.14	−0.01	−0.05	−0.19	−0.07	0.15	0.18	0.15	0.11	0.10	−0.27	−0.22	−0.20	−0.17	−0.37	−0.33	−0.34	−0.54	0.45	

Among demographic variables, age was most strongly correlated with vaccine hesitancy, with younger people being more vaccine hesitant (*r*_s_ = −0.30). Unsurprisingly, age was also negatively correlated with informational use of social media (especially Instagram, *r*_s_ = −0.35, Twitter, *r*_s_ = −0.32 and YouTube, *r*_s_ = −0.30), which was in turn positively correlated with conspiracy suspicions (especially with regards to Facebook, *r*_s_ = 0.25, YouTube, *r*_*s*_ = 0.24 and Instagram, *r*_s_ = 0.21).

Membership of an other than white ethnic group was positively correlated with vaccine hesitancy (*r*_s_ = 0.14), as was female gender (*r*_s_ = 0.08). Level of education was positively correlated with attitudes towards vaccines in general (*r*_s_ = 0.16) and negatively correlated with coronavirus conspiracy suspicions (*r*_s_ = −0.15), and so it is perhaps surprising that it should be uncorrelated with coronavirus vaccine hesitancy (*r*_s_ = −0.01); on the other hand, it is negatively correlated with age (*r*_s_ = −0.23), which could act to mask a relationship. Household income was found to have a very weak negative correlation with vaccine hesitancy (*r*_s_ = −0.05), but also to have a slightly stronger negative correlation with all forms of risk perception (*r*_s_ ⊂ [−0.07, −0.08]).

Given the high fatality rate for COVID-19 among members of certain minority ethnic groups in the UK (ONS, 2020), it is noteworthy that ethnicity was uncorrelated with perceived risk to the self (*r*_s_ = 0.00). Ethnic minority status was, however, positively correlated both with conspiracy suspicions (*r*_s_ = 0.11) and with use of social media for information about coronavirus (especially YouTube, *r*_s_ = 0.19, and WhatsApp, *r*_s_ = 0.20), and negatively correlated with both vaccine attitudes (*r*_s_ = −0.09) and age (*r*_s_ = −0.20). It is thus already apparent from the correlation matrix that there are a number of variables which might account for the association between ethnicity and vaccine hesitancy. Given suggestions that higher vaccine hesitancy among minority ethnic groups might be accounted for by mistrust, it is interesting that there was no correlation between ethnicity and trust in government (*r*_s_ = 0.00), a very weak correlation between membership of an other than white ethnic group and trust in scientists, whether working in the academic or the private sector (*r*_s_ = −0.05), and an only slightly stronger negative correlation with trust in medical professionals (*r*_s_ = −0.07).

### Statistical models and hypothesis tests

**Fig. 2.**
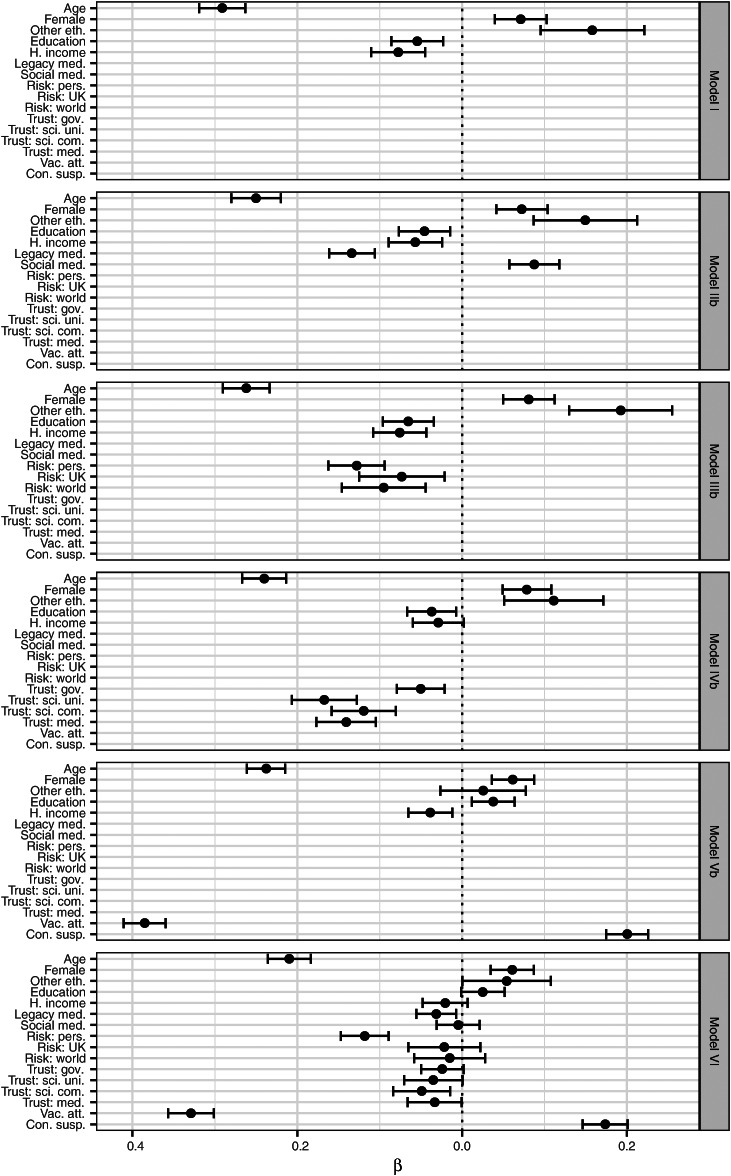
Predictors of vaccine hesitancy, with 95% confidence intervals.

Tables 4 and 5 present a series of eight linear rank-order models. Coefficients are quoted both as estimates with *p* values and as 95% confidence intervals, and visualised in Fig. 2 (which excludes models that do not feature demographic predictors).

**Table 4.** Linear rank-order models I, IIa, IIb, IIIa and IIIb

Term	Est.	s.e.	*t*	Low	High	*p*
Model I, *F*_5,3580_ = 101.81, *r*^2^ = 0.12, adj. *r*^2^ = 0.12						
(Intercept)	2390.30	99.67	23.98	2194.88	2585.73	<0.001
Age	−0.29	0.01	−20.33	−0.32	−0.26	<0.001
Female	0.07	0.02	4.45	0.04	0.10	<0.001
Other eth.	0.16	0.03	4.93	0.10	0.22	<0.001
Education	−0.05	0.02	−3.39	−0.09	−0.02	<0.001
H. income	−0.08	0.02	−4.65	−0.11	−0.04	<0.001
Model IIa, *F*_2,3941_ = 139.50, *r*^2^ = 0.07, adj. *r*^2^ = 0.07						
(Intercept)	1941.04	43.90	44.21	1854.96	2027.11	<0.001
Legacy med.	−0.16	0.01	−11.47	−0.19	−0.13	<0.001
Social med.	0.19	0.01	13.48	0.16	0.22	<0.001
Model IIb, *F*_7,3561_ = 89.73, *r*^2^ = 0.15, adj. *r*^2^ = 0.15						
(Intercept)	2359.32	108.49	21.75	2146.61	2572.04	<0.001
Age	−0.25	0.02	−16.34	−0.28	−0.22	<0.001
Female	0.07	0.02	4.59	0.04	0.10	<0.001
Other eth.	0.15	0.03	4.68	0.09	0.21	<0.001
Education	−0.05	0.02	−2.88	−0.08	−0.01	0.004
H. income	−0.06	0.02	−3.42	−0.09	−0.02	<0.001
Legacy med.	−0.13	0.01	−9.44	−0.16	−0.11	<0.001
Social med.	0.09	0.02	5.69	0.06	0.12	<0.001
Model IIIa, *F*_3,3711_ = 114.85, *r*^2^ = 0.08, adj. *r*^2^ = 0.08						
(Intercept)	2704.86	43.08	62.79	2620.39	2789.32	<0.001
Risk: pers.	−0.20	0.02	−11.75	−0.24	−0.17	<0.001
Risk: UK	−0.04	0.03	−1.68	−0.10	0.01	0.093
Risk: world	−0.09	0.03	−3.47	−0.14	−0.04	<0.001
Model IIIb, *F*_8,3369_ = 98.55, *r*^2^ = 0.19, adj. *r*^2^ = 0.19						
(Intercept)	2861.03	103.96	27.52	2657.20	3064.86	<0.001
Age	−0.26	0.01	−18.12	−0.29	−0.23	<0.001
Female	0.08	0.02	5.09	0.05	0.11	<0.001
Other eth.	0.19	0.03	6.04	0.13	0.26	<0.001
Education	−0.07	0.02	−4.13	−0.10	−0.03	<0.001
H. income	−0.08	0.02	−4.58	−0.11	−0.04	<0.001
Risk: pers.	−0.13	0.02	−7.34	−0.16	−0.09	<0.001
Risk: UK	−0.07	0.03	−2.76	−0.12	−0.02	0.006
Risk: world	−0.10	0.03	−3.67	−0.15	−0.04	<0.001

**Table 5.** Linear rank-order models IVa, IVb, Va, Vb and VI

Term	Est.	s.e.	*t*	Low	High	*p*
Model IVa, *F*_4,3759_ = 201.04, *r*^2^ = 0.18, adj. *r*^2^ = 0.18						
(Intercept)	3144.73	46.51	67.62	3053.55	3235.91	<0.001
Trust: gov.	−0.06	0.01	−3.91	−0.09	−0.03	<0.001
Trust: sci. uni.	−0.17	0.02	−8.64	−0.21	−0.13	<0.001
Trust: sci. com.	−0.13	0.02	−6.66	−0.17	−0.09	<0.001
Trust: med.	−0.18	0.02	−9.63	−0.21	−0.14	<0.001
Model IVb, *F*_9,3411_ = 131.29, *r*^2^ = 0.26, adj. *r*^2^ = 0.26						
(Intercept)	3250.20	102.05	31.85	3050.12	3450.29	<0.001
Age	−0.24	0.01	−17.66	−0.27	−0.21	<0.001
Female	0.08	0.02	5.22	0.05	0.11	<0.001
Other eth.	0.11	0.03	3.63	0.05	0.17	<0.001
Education	−0.04	0.02	−2.44	−0.07	−0.01	0.015
H. income	−0.03	0.02	−1.82	−0.06	0.00	0.069
Trust: gov.	−0.05	0.01	−3.38	−0.08	−0.02	<0.001
Trust: sci. uni.	−0.17	0.02	−8.31	−0.21	−0.13	<0.001
Trust: sci. com.	−0.12	0.02	−6.01	−0.16	−0.08	<0.001
Trust: med.	−0.14	0.02	−7.63	−0.18	−0.10	<0.001
Model Va, *F*_2,3899_ = 1031.80, *r*^2^ = 0.35, adj. *r*^2^ = 0.35						
(Intercept)	2319.43	49.89	46.49	2221.62	2417.24	<0.001
Vac. att.	−0.38	0.01	−28.94	−0.41	−0.35	<0.001
Con. susp.	0.23	0.01	17.95	0.21	0.26	<0.001
Model Vb, *F*_7,3524_ = 374.49, *r*^2^ = 0.43, adj. *r*^2^ = 0.43						
(Intercept)	2726.33	94.53	28.84	2541.00	2911.67	<0.001
Age	−0.24	0.01	−20.13	−0.26	−0.21	<0.001
Female	0.06	0.01	4.73	0.04	0.09	<0.001
Other eth.	0.03	0.03	0.97	−0.03	0.08	0.333
Education	0.04	0.01	2.85	0.01	0.06	0.004
H. income	−0.04	0.01	−2.83	−0.07	−0.01	0.005
Vac. att.	−0.39	0.01	−29.53	−0.41	−0.36	<0.001
Con. susp.	0.20	0.01	15.41	0.17	0.23	<0.001
Model VI, *F*_16,3198_ = 170.11, *r*^2^ = 0.46, adj. *r*^2^ = 0.46						
(Intercept)	3235.30	108.18	29.91	3023.20	3447.40	<0.001
Age	−0.21	0.01	−15.79	−0.24	−0.18	<0.001
Female	0.06	0.01	4.57	0.03	0.09	<0.001
Other eth.	0.05	0.03	1.98	0.00	0.11	0.048
Education	0.03	0.01	1.87	0.00	0.05	0.062
H. income	−0.02	0.01	−1.47	−0.05	0.01	0.140
Legacy med.	−0.03	0.01	−2.54	−0.06	−0.01	0.011
Social med.	0.00	0.01	−0.36	−0.03	0.02	0.721
Risk: pers.	−0.12	0.01	−8.02	−0.15	−0.09	<0.001
Risk: UK	−0.02	0.02	−0.96	−0.07	0.02	0.336
Risk: world	−0.02	0.02	−0.68	−0.06	0.03	0.493
Trust: gov.	−0.02	0.01	−1.81	−0.05	0.00	0.071
Trust: sci. uni.	−0.03	0.02	−1.93	−0.07	0.00	0.054
Trust: sci. com.	−0.05	0.02	−2.78	−0.08	−0.01	0.006
Trust: med.	−0.03	0.02	−2.00	−0.07	0.00	0.046
Vac. att.	−0.33	0.01	−23.17	−0.36	−0.30	<0.001
Con. susp.	0.17	0.01	12.42	0.15	0.20	<0.001

Model I tests demographic predictors. Importantly, level of education emerges as a predictor of lower vaccine hesitancy once other demographic variables are controlled for. H1 (negative association with age), H2 (positive association with female gender), H3 (positive association with other than white ethnic group) and H5 (negative association with household income) are all supported, *p* < 0.001, as is H4 (negative association with level of education), *p* < 0.001. Relatively, little variation in vaccine hesitancy is explained by demographic variables alone (*r*^2^ =  0.12), although, as we shall see, these explain more than some non-demographic variables. Moreover, the coefficients for age and female gender remain almost unchanged throughout the remaining models.

Model IIa tests information source predictors, with model IIb adding demographic controls. These show that the association of legacy media with lower vaccine hesitancy and the association of social media with higher vaccine hesitancy are independent and only partially explained by age. H6 (negative association with legacy media) and H7 (positive association with social media) are supported, *p* < 0.001. However, without demographic controls, these variables explain less variance in vaccine hesitancy than demographic variables alone (*r*^2^ = 0.07 for model IIa).

Model IIIa tests risk perception predictors, with model IIIb adding demographic controls. These show that perception of personal risk is the strongest predictor, although its association is partially explained by demographic variables (note the positive correlation between age and perceived personal risk, *r*_s_ = 0.22). H8a (positive association with perceived personal risk) is supported both before and after controls, *p* < 0.001. H8b (positive association with perceived risk to other people in the UK) is unsupported before controls, *p* = 0.093, but supported after controls, *p* = 0.006. H8c (positive association with perceived risk to people elsewhere in the world) is supported both before controls, *p* < 0.001, and after controls, *p* < 0.001. However, without demographic controls, these variables still explain less variance in vaccine hesitancy than demographic variables alone (*r*^2^ =  0.08 for model IIIa).

Model IVa tests trust predictors, with model IVb adding demographic controls. All tested forms of trust are significant in model IV, *p* < 0.001, but trust in the government had by far the smallest effect, whereas trust in scientists had the largest. Significance and effect sizes were virtually unchanged by controls. H9a–d are therefore all supported, both before and after demographic controls. It is notable that the association of other than white ethnic group with vaccine hesitancy (e.g. in model I) is partially reduced by introduction of trust variables as predictors in model IVb.

Model Va tests vaccine attitude and conspiracy suspicion predictors, with model Vb adding demographic controls. H10 (negative association with vaccine attitudes) and H11 (positive association with conspiracy suspicions) are supported both before and after controls, *p* ≤ 0.001. Model Va explains substantially more of the variance in observed vaccine hesitancy than any previous model (*r*^2^ = 0.35 as opposed to *r*^2^ =  0.19 for model IIIb and *r*^2^ = 0.26 for model IVb), and this rises further still with demographic controls in model Vb (*r*^2^ = 0.43). It is notable that the association between vaccine hesitancy and ethnic minority membership is statistically insignificant in model Vb but not in model IVb, which suggests that more of the variance associated with ethnicity is explained by vaccine attitudes and conspiracy suspicions than by trust. Moreover, the negative association between vaccine hesitancy and education in models I, IIb, IIIb and IVc is replaced by a positive association in model Vb.

Model VI tests all predictors together. It can be observed that model VI represents only a very marginal improvement over model Vb in terms of variation explained (*r*^2^ = 0.46 as opposed to *r*^2^ = 0.43). In other words, if we already know a person's age, gender, income, education, attitudes to vaccines in general and suspicions with regards to the possibility of a coronavirus-related conspiracy, then we will be able to predict his or her level of coronavirus vaccine hesitancy quite well, and additionally knowing his or her ethnic group, perception of coronavirus risks, level of trust in scientists, medics, and the government and sources of coronavirus information will tell us little more. It is also noteworthy that the coefficients both for conspiracy suspicions and for vaccine attitudes change very little between models Va, Vb and VI, while all forms of risk perception but perceived personal risk disappear from model VI, as do the effects of social media and most of the effects of trust. The positive association with education from model Vb remains, but loses statistical significance.

As discussed above, logit models were constructed because of theoretical concerns regarding type I errors. These concerns do not appear to have been borne out. Looking across all coefficients in all models, 44 were found to be significant at *p* < 0.001 in the linear rank-order models, whereas 45 were found to be significant to the same level in the logit models. This means that there is no evidence of type I error inflation as a result of the use of linear rank-order models.

## Conclusions and recommendations

As expected given findings of earlier studies, vaccine hesitancy was found to be negatively associated with age, household income, level of education, level of coronavirus risk perception (possibly excluding perceived risk to people living in other countries), trust in government, scientific and medical authorities and informational reliance on legacy media, and positively associated with female gender, other than white ethnic group, and informational reliance on social media. The effect associated with age was consistent across models, as was the weaker (but still very highly statistically significant) effect associated with gender, indicating that these effects are not explicable in terms of mutual correlations with other variables measured here. However, the most powerful predictors of vaccine hesitancy were attitudes to vaccines in general and conspiracy suspicions with regards to coronavirus, which appear to explain much more of the variation in coronavirus vaccine hesitancy than any of the aforementioned variables. Indeed, much or all of the variation associated with ethnic group, trust and informational reliance on social media appears to be explained by these two variables, whereas the association with education is reversed once they are controlled for.

It is notable that the association of other than white ethnic group with vaccine hesitancy (e.g. in model I) is partially reduced by introduction of trust variables as predictors in model IVb. This suggests that some of the heightened vaccine hesitancy which has been observed in some minority ethnic groups is attributable to lower scientific or medical trust. However, these variables were only weakly correlated with ethnicity, and government trust was not correlated at all. Ethnicity loses much more of its predictive power after controls for conspiracy suspicions and vaccine attitudes than it does after controls for trust, which suggests that these suspicions and attitudes may account for more of the vaccine hesitancy that is associated with minority ethnic group membership. However, this does not mean that a general intervention designed to address vaccine attitudes and conspiracy suspicions in the population as a whole would remove the association between vaccine hesitancy and ethnicity: as Chou and Budenz caution, health communicators should eschew generic messaging (2020). Useful guidelines exist for developing vaccination campaigns targeting specific ethnic or religious groups (Butler, MacDonald & Sage Working Group on Vaccine Hesitancy 2015).

The findings of this study suggest that vaccine hesitancy is strongly tied to both conspiracy suspicions and attitudes to vaccines in general. This presents a unique challenge for public health organisations and government vaccination programmes, which need to combat misinformation to ensure uptake of COVID-19 vaccination. Although the findings presented here cannot support the idea of a causal link with media usage, they are consistent with the assumption of such a link, which is also consistent with or supported by a large volume of research literature. Lower levels of vaccine hesitancy among respondents who rely on legacy media for information about coronavirus are consistent with the assumption that certain forms of communication may already be having a positive effect, even if they are not necessarily taking place through channels used by all members of the population. By contrast, the continued association of social media use with vaccine hesitancy (even after demographic controls) is consistent with the assumption that platform attempts to promote the dissemination of valid information and limit the dissemination of misinformation (e.g. UK Government, 2020) have not achieved an impact – or had not by the time of data collection. Moreover, the finding that the predictive power of social media use disappears with controls for coronavirus conspiracy suspicions and general vaccine attitudes is consistent with the assumption that the association of social media with vaccine hesitancy may be attributable to misinformation, which would provide grounds for optimism that interventions designed to limit the circulation of misinformation on those platforms may ultimately bear fruit.

## Limitations and scope for further research

The research here relies entirely on self-report measures for dependent and independent variables. Moreover, data collection was carried out as the rollout of the UK's vaccination programme began, and before several vaccines had been approved. As noted above, this was only shortly after several major platforms adopted enhanced policies to limit the circulation of coronavirus disinformation. Finally, although the sample was designed to be representative of the UK population (and appears representative on key demographic variables), it is not a true random sample.

Although this study has largely confirmed expectations arising from earlier studies and clarified the relative importance of the hypothesised predictors, an approach such as mediation analysis may be needed in order to further explore the inter-relations between the variables in question. Moreover, further data collection is urgently required in order to understand the reasons for different vaccine attitudes and levels of conspiracy suspicions within minority ethnic communities, particularly given that this does not appear to be accounted for by lower medical and political trust. The finding that the effects of age were not reducible to risk perceptions, conspiracy suspicions, vaccine attitudes or media use also needs further investigation, as does the finding that education is positively associated with vaccine hesitancy after controls for vaccine attitudes and coronavirus conspiracy suspicions. Finally, more research is clearly needed in order to understand the basis of vaccine hesitancy among women, as the association of vaccine hesitancy with female gender is not explained by any of the other variables investigated here. The effect size detected here appears relatively small, but existing research suggests that female caregivers may have an especially important role in vaccination decision-making within many communities (Allen et al., 2012, p. 3). If this holds true for coronavirus vaccination, then women will be a particularly important audience to reach, as vaccine hesitance on the part of one individual might potentially result in vaccine non-uptake throughout an entire family unit.

## Technical appendix

All calculations were carried out in R 3.6.3 (R Core Team, 2020), with psych 2.0.12 (Revelle, 2020) for calculation of Guttman's lambda 6. Visualisations were created using ggplot2 3.3.3 (see Wickham, 2016).

## Footnotes

† The notes appear after the main text.

## Note

1 Due to rounding, percentages are not guaranteed to sum to 100.
